# Cell-engineered technologies for wound healing and tissue regeneration

**DOI:** 10.1038/s44385-025-00042-w

**Published:** 2025-10-24

**Authors:** Malay Nayak, Durba Banerjee, Vangala Venugopal, Susheel Kumar Nethi, Ayan Kumar Barui, Sudip Mukherjee

**Affiliations:** 1 School of Biomedical Engineering, IIT (BHU), Varanasi, Uttar Pradesh India; 2 Department of Pharmacology, Penn State College of Medicine, Hershey, PA USA; 3 Department of Chemical and Biological Engineering, Iowa State University, Ames, IA USA; 4 Department of Basic Sciences, School of Sciences and Humanities, SR University, Warangal, Telangana India

**Keywords:** Biological techniques, Stem cells, Diseases, Medical research

## Abstract

This review provides a comprehensive analysis of diverse cell-engineered technologies for wound healing and tissue regeneration, highlighting various engineered techniques in a single article. It discusses different types of genetic modifications in various cell types to enhance cellular therapeutic properties. It also explores innovative cell delivery systems, including hydrogels and 3D bioprinting. Additionally, we evaluate the clinical applicability of these technologies and highlight key challenges, providing a future research direction.

## Introduction

Wound healing is an intricate and vibrant biological process that repairs injured or damaged tissues^1^. It involves dynamic interaction between molecular, cellular, and biochemical processes that move forward through a sequential step, including hemostasis, inflammation, proliferation, and remodeling^2^. These events are synchronized by various cell types and biomolecules that work together for wound closure and to regenerate injured tissue^3^. Any irregularities in this cascade of events can contribute to critical non-healing conditions, a substantial concern in clinical therapy, especially for conditions like diabetes, vascular ischemic diseases, and immunosuppression^4^. Wound healing is required to protect the body from infection, limit tissue damage, and restore tissue function.

**Fig. 1:**
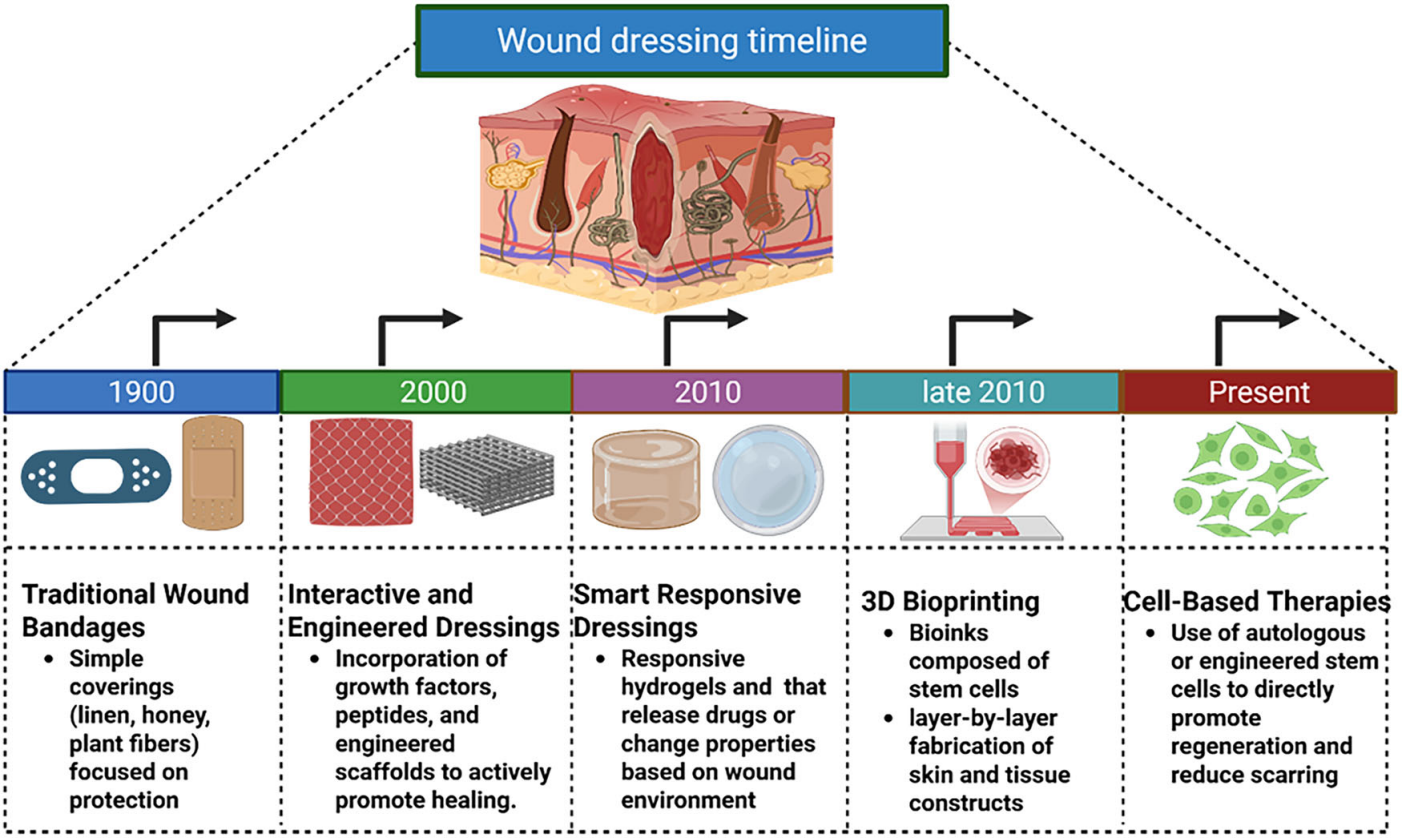
Timeline of wound dressing technologies.

Chronic wounds impact millions of people worldwide and place a heavy financial strain on healthcare systems, making them a major global health concern. For instance, it is estimated that the United States spends over $149 billion, China spends $43 billion, and India spends approximately $3 billion annually on wound care^5^. The global wound care market is expected to reach over $29.6 billion by 2030, having produced about $22.25 billion in 2023. The largest and fastest-growing section of the market is advanced wound dressings, which were valued at about $7.75 billion in 2023. Globally, the cost of advanced wound dressings varies from as little as $0.03 for simple dressings to more than $26 for specialty collagen-based dressings^6^. While researchers have gained a thorough understanding of the biological processes underlying wound healing, the conventional treatments, including topical applications and dressings, often provide insufficient care for intense wounds^7^. Hence, there is a growing demand for innovative therapies, including biomaterials, growth factors, and cell-based approaches, which actively promote tissue regeneration and improve healing effects. The evolution of wound healing strategies has progressed from simple protective coverings to sophisticated regenerative approaches. The latest methods focus on integrating delivery systems, biomaterial scaffolds, and genetic modification to maximize cell integration, survival, and function at wound sites. This strategy aims to not only close wounds but also minimize scarring and restore skin function. Burns, non-healing wounds that don’t heal, and chronic diabetic ulcers are most affected by these advancements. Furthermore, new developments in biofabrication methods, such as 3D bioprinting, allow for the accurate creation of skin substitutes by combining various cell types, thereby increasing the therapeutic potential. Recent studies show a trend toward individualized, multidimensional cell-engineered treatments that greatly improve healing dynamics, with a particular emphasis on scalability and clinical translation. A significant development in wound care paradigms is represented by the changing landscape of cell-based therapy. This review explores the advancements in cell-engineered technologies that are transforming conventional therapeutic methods, moving towards more effective and personalized strategies for tissue repair. This progression, from traditional bandages to cutting-edge cell-based therapies, is visually summarized in Fig. 1.

Cell-engineered technologies have emerged as a potential frontier in tissue engineering and regenerative medicine, specifically for wound healing applications^8^. This review aims to comprehensively explore the role of various cell types in wound healing, assess the advancements in engineered cell-based therapies for tissue regeneration in detail, and highlight cutting-edge techniques that enhance therapeutic efficacy. It further evaluates clinical applications, outcomes, and current limitations of these therapies, while providing insights into ongoing challenges and potential future directions in research and translational development.

## Role of cells in wound healing

The process of wound healing is facilitated by coordination between multiple cell types. Of these cell types, keratinocytes, fibroblasts, and endothelial cells play essential roles in wound repair and regeneration. Within the wound microenvironment, these cell types interact and orchestrate the cascade of different events during different healing phases, ultimately culminating in the restoration of skin structure and function^9^.

### Fibroblasts

Fibroblasts are located in the dermal layer of the skin and play a key role in the proliferative and remodeling phases of wound healing. Fibroblasts get activated upon injury and migrate into the wound area, producing extracellular matrix proteins such as fibronectin, collagen, and proteoglycans^10^. This extracellular matrix offers fundamental support for regenerating tissue as a scaffold for cell migration. On the other hand, the collagen of the ECM, with the help of integrin, regulates the function of fibroblasts by providing mechanical cues and by activating integrin signal pathways^11^. The activated fibroblasts that express alpha-smooth muscle actin (α-SMA) differentiate into myofibroblasts. These myofibroblasts exercise contractile forces that facilitate wound contraction and thereby reduce the size of the wound^12^. Additionally, fibroblasts release growth factors and cytokines, including platelet-derived growth factor (PDGF) and transforming growth factor-beta (TGF-β), which further influence other cells that guide wound healing. In chronic wounds that do not heal, these fibroblasts usually show irregular behavior characterized by abrogated migration, impaired ECM production, and senescence^13^. These aberrations delay prompt wound closure and have been targeted by therapeutics, which also include transplanting fibroblast-conditioned media of healthy autologous fibroblasts^14,15^.

### Keratinocytes

The keratinocytes are the primary cell type present in the epidermis that are essential for wound surface-epithelialization. After an injury, keratinocytes around the wound undergo a phenotypic switch that allows them to proliferate, migrate, and ultimately differentiate to restore the skin barrier^16^. This keratinocyte migration is influenced by ECM components and a variety of growth factors such as epidermal growth factor (EGF), keratinocyte growth factor (KGF), and laminin. The process of re-epithelialization includes several key steps: basal keratinocytes migrate to cover the wound bed, proliferate to expand the cell population, and stratify to reform the multilayered epidermis^17^. The efficiency and pace of this process are important for stopping infection and blood loss from the wound site. In recent years, keratinocyte-based dressings and cultured keratinocyte sheets have been explored as cell therapy products for burn wounds and chronic ulcers. Keratinocytes can be sourced from either autogenic or allogenic origins. In the case of autogenic sourcing, the cells are collected from a patient’s uninjured area^18^. For allogenic sourcing, various immortalized keratinocyte cell lines, such as NIKS® and human epidermal keratinocyte cell lines, are cultured^19,20^. Additionally, cells can also be isolated and cultured from donated human foreskin tissue. These cell therapies intend to drive wound closure and restore the skin barrier function^21^.

### Endothelial cells

Angiogenesis, the formation of new blood vessels from existing vasculature, is key for wound healing^22^. Endothelial cells are located as the inner lining of the blood vessels and are key to the angiogenesis process during wound healing. Angiogenesis is evident to supply nutrients, oxygen, and immune cells to healing tissue. Endothelial cells are activated by the hypoxic conditions at the damaged tissues, and growth factors, including fibroblast growth factor-2 (FGF-2) and vascular endothelial growth factor (VEGF), promote their endothelial cell proliferation, migration, and tube formation^23^. After the establishment of these capillary networks, endothelial cells are responsible for maintaining vascular integrity and regulating the inflammatory response. Dysfunctional angiogenesis, as seen in diabetic wounds, results in poor tissue perfusion and delayed healing. Therapeutic strategies aimed at enhancing angiogenesis often involve the delivery of endothelial progenitor cells (EPCs) or VEGF-producing cells^24^. These approaches have shown promise in preclinical models, though clinical translation is ongoing.

## Overview of cell engineering techniques for tissue regeneration: advantages and disadvantages

Engineered cell-based therapies are treatments that use living cells precisely modified in vitro—through genetic, biomaterial, or biochemical methods—to enhance their therapeutic function for repairing tissues and treating diseases such as cancer, genetic disorders, and immune conditions. Table 1 provides a comparative overview of the main cell engineering techniques discussed for wound healing and tissue regeneration in the review. It highlights how these advanced approaches—including genetic modification, differentiation protocols, scaffold integration, and engineered cell constructs—offer unique benefits such as enhanced healing, improved angiogenesis, or immune modulation.

**Table 1:** Various engineered techniques for tissue regeneration using cellular engineering

Cell engineering technique	Description	Advantages	Disadvantages
Genetic Engineering with CRISPR/Cas9	Modification of genes (e.g., knock-out of Integrin β1 in iPSCs)^49^	Enhanced specific cellular functions (e.g., migration, angiogenesis); precise, targeted changes	Risk of off-target effects; potential immune responses; regulatory and safety concerns
Differentiation of iPSCs to Desired Cell Types	Directing iPSCs to become keratinocytes, endothelial cells, etc^51^.	Unlimited proliferation; potential for autologous, patient-specific therapy; broad applicability	Tumorigenicity risk; complex protocols; regulatory hurdles
Genetically Engineered Growth Factor Expression	Engineering fibroblasts to overexpress genes like VEGF165^57^	Boosts angiogenesis, accelerates early wound closure; controlled, sustained growth factor delivery	Benefit may be limited to early healing phases; possible safety issues; scalability
Use of Immortalized Cell Lines (e.g., SV40 T antigen)	Introduction of genes that allow cells to proliferate indefinitely (e.g., reversibly immortalized keratinocytes)^58^	Overcomes primary cell senescence; consistent cell supply; retains non-tumorigenic properties	Genetic modification concerns; potential oncogenicity if not well controlled
Pre-vascularized 3D Cell Constructs with Hydrogels/Scaffolds	Combining engineered cells (e.g., hiPSC-derived vascular cells + ECFCs) in biomaterial matrices for implantation^50^	Improved engraftment; facilitates angiogenesis; allows physiological tissue structure; customizable	Manufacturing complexity; integration challenges; higher cost
Genetic Modification of MSCs	Engineered umbilical cord-derived MSCs to overexpress angiopoietin-1 (ANG1)^44^ or Gremlin1^43^ or CCR2 receptor^45^	Improved MSC survival and therapeutic efficacy, promoting angiogenesis, modulating immune responses, and accelerating diabetic wound healing.	Regulatory issues
Enucleated Mesenchymal Stem Cells (Cargocytes)	Created nucleus-free mesenchymal stem cells (BMSCs) that carry an aptamer for targeted binding and are loaded with IL-4 mRNA for sustained secretion^42^.	Acted as a targeted anti-inflammatory factory, inhibited NETs, and accelerated wound healing in diabetic models.	Viability was limited as the cells gradually disappeared within seven days, indicating a short-term therapeutic window.

### Types of engineered cell-based therapies for tissue regeneration

**Fig. 2:**
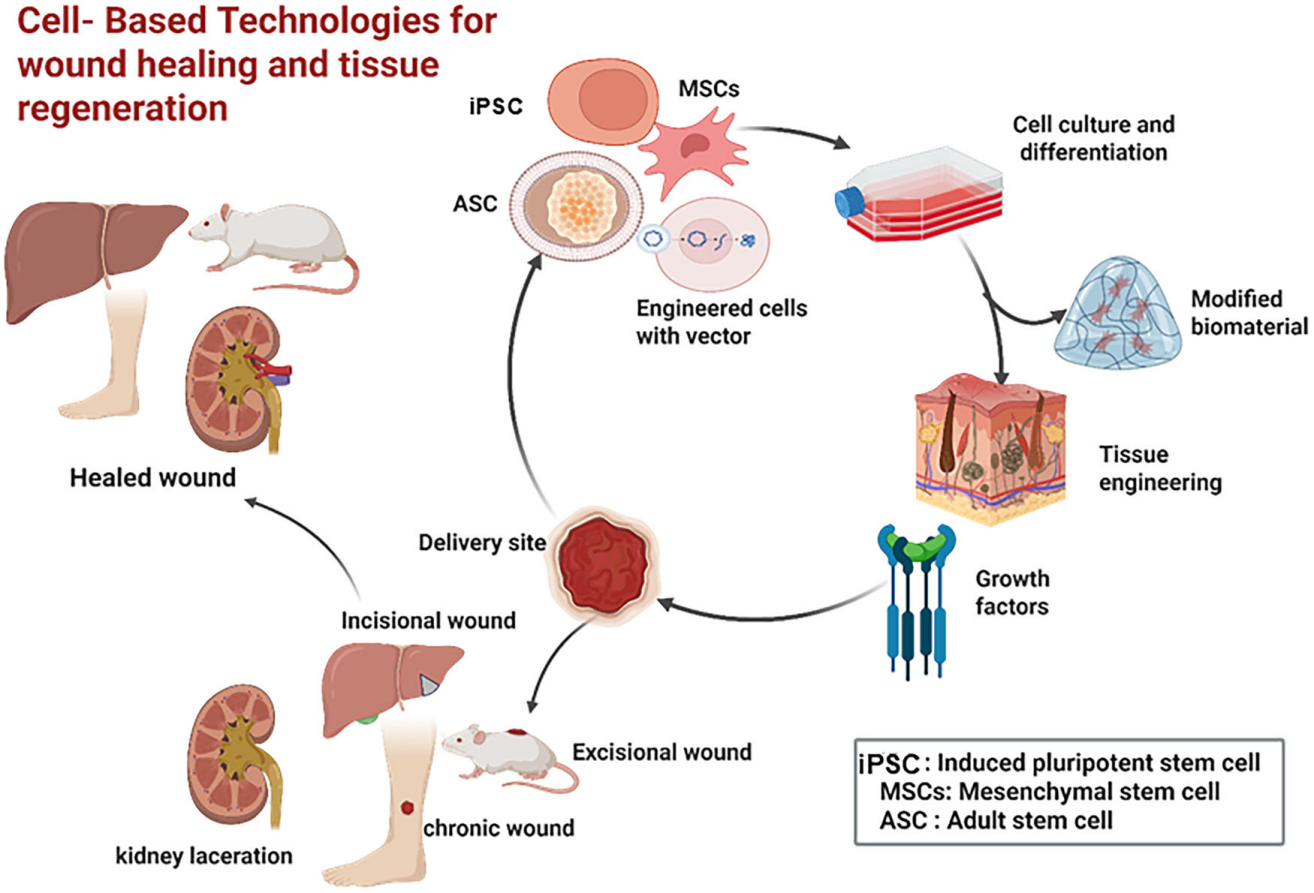
The comprehensive workflow of cell-based technologies for wound healing and tissue regeneration.

Advancements in cell-based therapies are rapidly transforming the landscape of wound healing and tissue regeneration. As demonstrated in Fig. 2, the process covers selecting from a wide array of cellular sources, applying targeted engineering strategies, integrating cells with supportive biomaterials, and ultimately utilizing these constructs for the repair of various wound types. This comprehensive approach streamlines the pathway from cell sourcing and modification to effective tissue restoration. In this section, we’ll explore fascinating approaches to engineering cells in innovative ways, highlighting their pivotal role in the exciting field of regenerative medicine.c

### Stem cell therapies and engineering for tissue regeneration

Stem cells are fundamental cells in the human body capable of developing into over 200 different cell types^25^. They are non-specialized cells that can transform into specialized cells, possessing the ability to self-renew, differentiate into various cell types, and proliferate. Stem cell therapies represent a cornerstone of regenerative medicine, offering unparalleled potential for restoring damaged tissues and promoting wound healing. A diverse array of stem cell types—including embryonic stem cells (ESCs), induced pluripotent stem cells (iPSCs), mesenchymal stem cells (MSCs), hematopoietic stem cells (HSCs), and tissue-specific adult stem cells—have been investigated for their regenerative capabilities^26^. These cells contribute to tissue repair not only through direct differentiation into target cell types but also via the secretion of trophic factors that modulate inflammation, stimulate angiogenesis, and facilitate extracellular matrix remodeling engineering. These Stem cells will further enhance the properties of stem cells towards tissue regeneration and wound healing. Collectively, stem cell–based approaches are redefining therapeutic strategies for chronic wounds, degenerative diseases, and complex tissue injuries, underscoring their pivotal role in the future of personalized and regenerative medicine.

#### Mesenchymal stem cells (MSCs)

Mesenchymal stem cells (MSCs), derived from bone marrow (BM-MSCs), adipose tissue (AT-MSCs), umbilical cord(UC-MSCs), or amniotic membrane (AM-MSCs), have emerged as pivotal agents in regenerative medicine due to their immunomodulatory, angiogenic, and anti-fibrotic properties^27^. Among the various sources, Bone Marrow-Derived Mesenchymal Stem Cells (BM-MSCs) are a well-studied type, capable of differentiating into various cell lineages, including bone, cartilage, and fat cells. They have a superior capacity for osteogenic (bone) and chondrogenic (cartilage) differentiation. This makes them the top choice for treating bone fractures, non-unions, and cartilage defects^28^. However, the procedure to harvest them is invasive, and their potency and number can decline with a donor’s age. While comparing different origins of MSCs, it has been observed that UC-MSCs show a higher proliferation rate, higher anti-inflammatory effect, greater clonality, and retardation of senescence than others^29,30^. In contrast, AT-MSCs are known for their high proliferation rates and strong angiogenic and immunomodulatory properties. They are particularly effective for soft tissue regeneration and wound healing, where improving blood supply and reducing inflammation are crucial^31^. Their regenerative capabilities stem from their innate ability to modulate immune responses, stimulate angiogenesis, and promote tissue repair, making them particularly well-suited for wound-healing applications. Naturally derived MSCs have already demonstrated significant therapeutic potential for tissue regeneration, with different types showing unique strengths. Clinical studies confirm that BM-MSCs accelerate wound healing by releasing proangiogenic factors and differentiating into critical cell types, as seen in patients with chronic leg ulcers, where studies by Falanga et al^32^. and Badiavas et al^33^. showed reduced wound size and complete wound closure. AD-MSCs promote enhanced wound healing, improved cosmesis, and increased vascularization through the secretion of a wide range of pro-healing factors like VEGF and TGF-β^34–38^. Furthermore, hUC-MSCs are gaining recognition for their ability to promote ulcer closure and symptom alleviation in conditions like diabetic foot ulcers^39,40^.

While these unengineered MSC strategies have proven effective, engineered MSCs are now more in demand because they offer a way to overcome the inherent limitations of naturally derived cells, such as inconsistent potency and survival rates, by enhancing their therapeutic properties and targeting capabilities. Xue et al. developed a sophisticated cellular engineering platform using novel lipid nanoparticles (LNPs) to reprogram adipose stem cells (ASCs) to accelerate Diabetic wound healing. They first synthesized and optimized a series of sugar alcohol-derived LNPs, identifying the DIM1T formulation as exceptionally efficient, surpassing even FDA-approved formulations and traditional methods like electroporation. A major challenge arose when attempting to achieve sustained protein expression using self-amplifying RNA (saRNA), as the ASCs’ natural immune response, specifically the PKR-eIF2α pathway, terminated translation within two days. To overcome this, the authors innovatively co-delivered the therapeutic saRNA with an mRNA encoding an immune-evasive E3 protein, which successfully blocked the immune response and enabled durable protein secretion for over nine days without altering the ASCs’ fundamental characteristics. This optimized LNP-saRNA/E3 mRNA complex was then used to engineer ASCs to secrete healing factors like CXCL12 and HGF (Hepatocyte Growth Factor), leading to the best result where these engineered cells significantly accelerated diabetic wound closure in mice, with all wounds treated by CXCL12-producing ASCs achieving complete closure by day 15. The primary limitation of this work, however, was the inherent immune-mediated translational shutdown of saRNA in ASCs, which necessitated the crucial addition of the E3 mRNA to achieve therapeutic efficacy^41^. In another study by Chen et al., they engineered enucleated mesenchymal stem cells (BMSCs), which they termed Cargocytes, to act as a targeted anti-inflammatory factory^42^. The primary engineering technique involved modifying these nucleus-free cells to carry an aptamer (DTA64) that specifically binds to the DEK protein on neutrophils, and loading them with Interleukin (IL)-4 mRNA for sustained secretion of the anti-inflammatory cytokine. This dual-action approach allowed the Cargocytes to actively seek out and reprogram the inflammatory cells at the wound site. The best result was that these Cargocytes, when delivered via an injectable hydrogel, significantly accelerated wound healing in diabetic models by inhibiting neutrophil extracellular traps (NETs) and reducing pro-inflammatory M1 macrophages. A key limitation, however, was that because the cells were enucleated, their viability was limited; they gradually disappeared from the wound microenvironment within seven days, indicating a short-term therapeutic window. Er Saw et al. engineered mesenchymal stem cells (MSCs) to improve their survival and therapeutic efficacy in a critical limb ischemia model. The primary cellular engineering technique involved the genetic modification of MSCs to overexpress Gremlin1 (Grem1), a protein that promotes angiogenesis and enhances cell tolerance to oxidative stress. They accomplished this by transducing the cells with a lentivirus carrying the Grem1 gene, thereby creating Grem1-MSCs. They load the cell with a hybrid hydrogel composed of type I collagen and melanin nanoparticles (MeNPs) to increase the cell survival and proliferation^43^. Deng et al. in their article, Engineered MSC derived from Umbilical cord to overexpress angiopoietin-1, which is a proangiogenic gene that helps in the maturation of new blood vessels. A lentiviral vector containing the CMV promoter with mouse ANG1 and GFP gene linked by the t2A linker was constructed^44^. Kuang et al. addressed the limitations of MSC-based therapies by genetically engineering MSCs to overexpress C–C motif chemokine receptor 2 (CCR2). This enhanced MSCs’ targeted migration and immunoregulatory potential in response to CCL2. In diabetic mouse models, CCR2-engineered MSCs (MSCsCCR2) showed improved homing to injured sites, inhibited monocyte infiltration, reshaped macrophage inflammatory properties, promoted regulatory T cell accumulation, and modulated systemic immune responses, ultimately accelerating diabetic wound healing. These findings present a novel strategy for genetically engineered MSCs to facilitate tissue repair in diabetic wounds^45^.

#### Induced pluripotent stem cell

Induced pluripotent stem cells (iPSCs), reprogrammed from somatic cells, offer an ethically unencumbered and scalable platform for wound healing by generating patient-specific therapeutic cell types such as mesenchymal stem cells (MSCs), keratinocytes, and fibroblasts^46^. iPSCs offer significant advantages over other stem cell types for wound healing because they can be easily harvested from a non-invasive source like a skin biopsy, have a virtually unlimited capacity for self-renewal, and can be differentiated into any cell type needed for specific therapeutic applications^47^. The main disadvantage of using iPSCs for wound healing is their teratogenic potential, which is the risk of forming tumors if the cells are not fully and properly differentiated before transplantation^48^. Advances in CRISPR editing and epigenetic resetting are refining differentiation efficiency, positioning iPSCs as a transformative tool for personalized, off-the-shelf regenerative solutions that address the multicellular complexity of chronic wounds^47^.

Ren et al. engineered induced pluripotent stem cells (iPSCs) to enhance their migratory and wound-healing abilities by using CRISPR-Cas9 gene editing to knock out the gene for Integrin β1 (Itgb1)^49^. This engineering technique gave the cells a significant advantage, as the resulting Itgb1-knockout iPSCs demonstrated decreased adhesion to the extracellular matrix (ECM) and a corresponding increase in their migratory capacity. This improved migration was crucial for accelerating wound healing, promoting angiogenesis, and enhancing blood perfusion in a skin wound model. Shen et al. aimed to create a robust and functional cell-based therapy for diabetic wounds by engineering a pre-vascularized construct. They used human-induced pluripotent stem cells (hiPSCs) as the foundation for their cellular therapy. The primary engineering technique was to differentiate these hiPSCs into early vascular cells (EVCs), a process that yields a mixture of endothelial cells and pericytes. They then combined these hiPSC-derived EVCs with endothelial colony-forming cells (ECFCs) from the peripheral blood of healthy donors. This combination of cells was then encapsulated in a hyaluronic acid (HA) hydrogel, which served as both a delivery vehicle and a supportive environment. This process allowed them to create a pre-formed, vascularized tissue construct in vitro before it was implanted into the diabetic wound^50^. In another study, Wu et al. engineered induced pluripotent stem cells (iPSCs) into keratinocytes by differentiating them with all-trans retinoic acid (RA) and BMP4. They tested these iPSC-derived keratinocytes in a deep second-degree burn wound model created on the abdomens of mice by applying a hot water-filled tube. The most significant finding was that the co-administration of the iPSC-derived keratinocytes with Hsp90α, a protein that promotes cell migration, led to a substantial acceleration of wound healing and enhanced re-epithelialization^51^.

### Fibroblast and keratinocyte cell engineering

Keratinocytes and fibroblasts synergistically drive wound healing through re-epithelialization, extracellular matrix (ECM) remodeling, and inflammation modulation. Keratinocytes migrate across wound beds, secreting epidermal growth factor (EGF) and transforming growth factor-β (TGF-β) to activate fibroblasts, which in turn produce collagen, fibronectin, and matrix metalloproteinases (MMPs) for ECM restructuring and wound contraction^52,53^. Clinically, Dermagraft—a cryopreserved dermal substitute of neonatal fibroblasts on a polyglactin scaffold—delivers fibroblasts that secrete collagen, VEGF, and FGF-2, achieving 30% complete closure in diabetic foot ulcers by 12 weeks (vs. 18% with standard care) through MMP balance and fibrosis reduction^54^. Conversely, Apligraf, a bilayered construct of fibroblasts in bovine collagen and keratinocytes, mimics native skin by reactivating dormant keratinocytes, correcting TGF-β/PDGF signaling, and suppressing scarring, with 89% healing in complex wounds and 94% closure in chronic cases^55^. These therapies target all healing phases: Dermagraft’s fibroblasts polarize macrophages to anti-inflammatory M2 phenotypes, lowering TNF-α and IL-6, while Apligraf’s keratinocytes accelerate epithelial migration via EGF, and its fibroblasts rebuild dermal structure^56^. Beyond direct cell application, strategies focusing on enhancing the wound microenvironment and cell functions are emerging by using genetically engineered cells. Shams et al. developed a novel approach to accelerate wound healing by genetically engineering human fibroblasts to overexpress the VEGF165 gene. Using a recombinant pcDNA3.1/Hygro (-) vector containing the CMV promoter, they successfully transfected the HuO2 fibroblast cell line by using the Turbofect transfection reagent. It showed a sevenfold increase in VEGF expression, confirmed by qRT-PCR and Western blotting. These modified cells were then integrated into a biocompatible polyurethane-cellulose acetate scaffold and applied to full-thickness wounds in a rat model. Their most significant finding was that this technique resulted in significantly enhanced angiogenesis and faster wound closure during the early stages of healing, although a key limitation observed was that final wound closure rates by day 15 were similar to all other groups, indicating the primary benefit of this technique lies in the initial, rapid phases of tissue repair^57^. In their study, Zhong et al. engineered cells by creating reversibly immortalized mouse keratinocytes (iKera) through the stable expression of the SV40 large T antigen (SV40 T). This technique provided a significant advantage by overcoming the limited lifespan of primary keratinocytes in culture. The reversibly immortalized cells retained long-term proliferative activity in vitro while remaining non-tumorigenic. They were then used in a proof-of-concept experiment, embedded in a citrate-based scaffold, to demonstrate that this combination provided more effective re-epithelialization and cutaneous wound healing than either the scaffold or the iKera cells alone^58^.

## Different methods of cell delivery

### Hydrogels and scaffolds

Hydrogels are a special type of biomaterial that can be employed as scaffolds for tissue regeneration and wound healing^59–61^. It is a crosslinked polymer with a three-dimensional structure that can absorb a large amount of fluid while maintaining its integrity. While hydrogels are hydrophilic, porous, 3D networks of crosslinked polymers, hydrogel scaffolds are precisely constructed hydrogels employed as constructs that promote cell attachment, growth, and differentiation, leading to tissue regeneration. The 3D networks of hydrogel scaffolds can mimic the extracellular matrix (ECM) of tissue, providing a supporting ecosystem for cellular growth^62^. Moreover, various hydrogels, including alginate and chitosan-based hydrogels, demonstrated great biocompatibility. Therefore, they often act as dressing materials, offering a humid and protective setting to the injured tissues. The hydrogel scaffolds are generally prepared employing different hydrophilic polymers (e.g., natural polymers: collagen, hyaluronic acid, chitosan, gelatin, alginate, fibrin, etc.; synthetic polymers: polyethylene glycol-PEG, polyvinyl alcohol-PVA, etc)^63^. Besides hydrogels, tissue engineering scaffolds can be fabricated using different other biomaterials, which include certain synthetic polymers (polylactic acid: PLA, polycaprolactone: PCL) as well as bio-ceramics (silicate-based ceramics, calcium-phosphate ceramics, etc). In one study, Kim et al. developed hyaluronic acid-modified human adipose tissue-derived ECM sheets (ECM-HA) and investigated their wound healing potential^64^. The results showed the efficient survival of adipose-derived stem cells seeded on the ECM-HA sheets. Lu et al. introduced a novel “living hydrogel” system comprising Lactococcus bacteria incorporated into a heparin-poloxamer thermoresponsive hydrogel. This system continuously produces VEGF to promote endothelial cell proliferation, migration, and tube formation, while also secreting lactic acid to induce an anti-inflammatory macrophage phenotype. This approach successfully enhanced angiogenesis in diabetic wounds and confined bacterial populations to the wound site, minimizing systemic toxicity^65^.

### 3D bioprinting techniques

3D bioprinting is a revolutionary technology in regenerative medicine^66,67^. These constructs resemble the structural and functional characteristics of natural tissues. The 3D bioprinting technology is highly significant in the fabrication of skin grafts as well as wound dressing, which leads to restoring injured tissues. While it is useful for wound healing, it also holds great potential in printing functional organs that could be employed for organ transplantation. Different 3D bioprinting techniques include extrusion-based, inkjet-based, laser-assisted, and stereolithography printing^68^.

In a report, Huang and colleagues reported that double-layer conductive skin can improve the wound healing process^69^. The authors employed 3D bioprinting-mediated techniques to fabricate the conductive skin scaffold with the use of a biomimetic bioink known as GHCM on a commercial extrusion-based bioprinter. The authors developed the double-layer (epidermal and vascularized dermal layers) skin scaffolds through the integration of fibroblasts (HFF-1), endothelial cells (HUVECs), and keratinocytes (HaCaTs). The results illustrated that the scaffolds offered a suitable conductive setting for cell signaling, cell proliferation, migration, and differentiation. This, in turn, facilitated faster re-epithelialization, vascularization of the injured skin, and collagen deposition, which are critical for regenerative medicine. In another study, Wang et al. demonstrated a unique microfluidic-supported bioprinting approach to fabricate microalgae-containing hollow fibrous photosynthetic scaffolds (MA-HF) medicine with the help of an extrusion-based 3 d printer and 365 nm UV crosslinking^70^. Oxygenic photosynthetic microalga Chlorella pyrenoidosa was combined with the scaffolds during 3D printing. Therefore, the scaffolds were capable of generating sustainable oxygen in the presence of light, which promoted cell growth, migration, and differentiation even in hypoxic environments. Additionally, the direct 3D printing of MA-HF into the wounds of a diabetic mouse model exhibited enhanced closure of wounds by ameliorating the hypoxic conditions in the injured tissues, promoting the angiogenesis process, and inducing collagen synthesis. The authors suggested that MA-HF could potentially be used for versatile tissue engineering applications. Zhang et al. also developed adipose-derived stem cells-laden 3D-printed skin scaffolds, composed of gelatin methacryloyl, hyaluronic acid methacryloyl, and adipose-derived extracellular matrix (adECM)^71^. Extrusion based 3 d printer was used with a thermos-controlled chamber, and photo crosslinking was done using 405 nm UV light. The administration of these 3D-printed bioactive skin scaffolds toward a full-thickness skin defect BALB/c nude mouse model exhibited faster wound healing compared to the control group by inducing angiogenesis, collagen deposition, and remodeling.

## Challenges and limitations in cell-based wound healing therapies

Despite significant progress, cell-based therapies for wound healing face critical hurdles, primarily concerning cell viability, immunogenic rejection of allogeneic cells, and complex regulatory landscapes. Overcoming these is crucial for clinical translation.

### Cell viability and retention issues

Post-transplantation, a hostile microenvironment leads to substantial therapeutic cell loss, with only 1–5% of intravenously delivered MSCs persisting at 72 h due to hypoxia, oxidative stress, inflammation, and mechanical trauma^72^. Strategies to improve survival include hypoxic preconditioning^73,74^, biomaterial scaffolds^75^, and genetic modification^76,77^. However, these introduce complexities in manufacturing and regulation. These unfavorable conditions can trigger apoptosis, necrosis, and impaired paracrine activity, sharply limiting the regenerative capacity of the therapy. In response, several strategies have been explored to enhance cell survival: hypoxic preconditioning aims to acclimatize cells to low-oxygen conditions and increase their resilience; genetic modifications target anti-apoptotic pathways or enhance trophic factor secretion to improve persistence and therapeutic efficacy; and, importantly, the application of biomaterial scaffolds—including hydrogels—represents a significant advancement in improving cell viability^78^. Hydrogels can be engineered to deliver nutrients, oxygen, and regulatory signals, release growth factors in a sustained manner, and even shield cells from immune attack. Advanced designs incorporate adhesive peptides or bioactive cues guiding cellular attachment, migration, and differentiation, further boosting the retention and paracrine activity of transplanted MSCs and other progenitor cells within the wound bed. Nevertheless, the multifaceted benefits of hydrogel-based and scaffold-assisted delivery systems in supporting cell viability, enhancing engraftment, and modulating the wound environment make them indispensable tools in the ongoing evolution of cell-based wound healing strategies^79,80^.

### Immunogenicity and rejection of allogeneic cells

Contrary to earlier beliefs, allogeneic cell therapies trigger innate and adaptive immune responses, reducing therapeutic durability^81^. This includes NK cell-mediated cytotoxicity^82^, T-cell activation^83^, and mononuclear phagocyte engagement^84^, leading to attenuated long-term efficacy compared to autologous cells^85^. Emerging mitigation strategies involve CRISPR/Cas9-mediated MHC ablation^86,87^, immune-isolating encapsulation, and short-term immunomodulation, though complete abrogation of immunogenicity remains elusive^88^. Data from rodent models have shown both monocyte/macrophage-mediated innate recognition and classic T and B cell-mediated adaptive responses against allogeneic cells, resulting in their rapid rejection. For example, mouse models have directly shown monocytes and macrophages act as critical sensors of allogeneic non-self, and their activation initiates acute rejection through differentiation into mature dendritic cells, production of pro-inflammatory cytokines (notably IL-12), and subsequent stimulation of T cell proliferation and IFN-γ production^89^. In non-human primates, such as rhesus macaques, administration of allogeneic mesenchymal stem cells (MSCs) led to the development of allo-specific antibodies, activation of cytotoxic lymphocytes, and elevated NK and B cell activity, all correlating with cell clearance^90^. These findings are paralleled by clinical observations in humans, where allogeneic cell therapies often show transient benefits and reduced engraftment due to similar immunological mechanisms. Collectively, both preclinical and clinical evidence establish that allogeneic cell therapies are subject to immune surveillance and clearance, underscoring the need to address immune compatibility to improve their therapeutic durability^91,92^. Recent immunoprotective hydrogels have shown promise in cell therapy by providing both antibacterial properties and immune modulation. These hydrogels act as physical and biological barriers, protecting transplanted cells from immune rejection while reducing infection^93^. By promoting anti-inflammatory responses and supporting tissue repair, they enhance the therapeutic durability of allogeneic cell therapies in wound healing.

### Regulatory considerations

The inherent biological complexity of cellular therapeutics poses unique regulatory challenges distinct from traditional drugs. Key impediments include cellular heterogeneity^94,95^, the development of reliable potency assays, and safety concerns like ectopic differentiation or tumorigenicity^96–98^. While frameworks like the FDA’s RMAT designation and EMA’s ATMP classification standardize requirements, interjurisdictional inconsistencies and a paucity of validated potency biomarkers continue to impede efficient clinical translation. A major, enduring challenge is the paucity of validated potency biomarkers and standardized functional assays applicable across different cell types and disease indications. Most cellular therapy developers, particularly small to medium enterprises or academic sponsors, face difficulty aligning their preclinical and clinical data with regulatory expectations, given limited resources and the evolving regulatory landscape. Additionally, the regulatory burden is compounded for combination products or those produced alongside devices, as multi-layered and overlapping legal requirements add significant operational complexity, especially in the European Union. While hospital exemptions and special exemptions may facilitate early clinical access, they raise concerns about potential gaps in safety and efficacy evaluation^99^.

Altogether, these multiple hurdles—biological variability, assay validation, interjurisdictional inconsistencies, and operational burden—continue to impede the efficient clinical translation of cell therapies, highlighting the urgent need for harmonization of standards and further development of robust, validated biomarkers and potency methods to ensure product safety, efficacy, and access for patients worldwide^100^. Interjurisdictional regulatory disparities pose significant challenges to the global clinical translation of cellular therapeutics by creating fragmented and inconsistent frameworks across different regions. Variations in product definitions, classifications, and approval pathways require developers to navigate distinct regulatory requirements in each jurisdiction, leading to increased complexity, cost, and development timelines. Differences in potency assay standards, preclinical safety evaluations, and clinical trial procedures further complicate multinational studies, often resulting in duplicated efforts and delayed trial initiations. Moreover, inconsistent post-approval surveillance and market access regulations can hinder streamlined patient availability worldwide^101^. These disparities collectively slow the advancement and equitable distribution of innovative cell therapies and highlight the urgent need for harmonized regulations and mutual recognition to facilitate more efficient and inclusive global clinical development^102^.

## Future directions in research and development

### Innovations in cell-delivery systems

The future of wound care hinges on breakthrough delivery systems that integrate sophisticated material design with real-time monitoring. New research is exploring innovative ways to combine sensors that monitor wounds with smart delivery systems that adjust treatment based on the wound’s changing condition. This approach helps provide the right therapy at the right time, improving healing and making treatment more effective. This next-generation approach, combining real-time biological sensing with programmable therapeutic release, represents a transformative leap beyond traditional treatments—enabling precisely orchestrated, spatially patterned delivery of cells and bio-factors.

In parallel, vascularized patterned hydrogels represent a game-changing approach to cell delivery. These engineered hydrogels contain perfusable channel networks that not only support embedded cells but also promote patterned vascularization once implanted, enhancing oxygen and nutrient distribution to the wound bed. This demonstrates that combining such hydrogels with cell therapy substantially improves vascular integration and tissue regeneration in vivo—a step toward bio-instructive scaffolds that support complex tissue healing never before realized^103^. Emerging technologies like injectable microneedle arrays and magnetically guided delivery enable minimally invasive, targeted transplantation. Together, these advances promise more effective, controlled, and personalized regenerative therapies.

### Potential for personalized medicine approaches

Personalized medicine will revolutionize chronic wound management by tailoring treatments to individual patient characteristics. Bioengineered skin substitutes, created with patient-specific cells, mimic natural skin, improving graft integration and reducing rejection^104^. Injectable hydrogels can be customized for controlled delivery of therapeutic agents for internal injuries^93^. Gene therapy, using liposomal or viral vectors for growth factor genes (PDGF, KGF), accelerates re-epithelialization, and topical siRNA therapy silences dysregulated genes^105^. Growth hormones (GHs) and GHRH agonists enhance collagen synthesis and regenerative capacity, particularly for elderly patients^106^. This integration of therapies promises faster recovery and reduced complications by addressing unique patient factors. Furthermore, advances in biomarker discovery and ‘omics’ technologies are enabling clinicians to stratify patients based on molecular profiles—such as their inflammatory status, proteomic wound biomarkers, and genetic predispositions—thereby guiding the selection of the most appropriate cellular or molecular therapeutic for each^107^. Comprehensive biomarker profiling using transcriptomics, proteomics, and metabolomics helps clinicians identify key determinants of healing or chronicity for each patient, such as pro-inflammatory cytokine levels, matrix metalloproteinase activity, and specific gene expression signatures^108^. This molecular insight allows for the stratification of patients, selecting those most likely to benefit from targeted therapies, whether that means enhanced angiogenesis, anti-fibrotic treatment, or immunomodulation^109^. Finally, future clinical models will likely combine these technologies via integrated digital health platforms that unify patient history, genomics, wound monitoring, and remote care. Such systems will support adaptive, individualized treatment plans with ongoing adjustment based on patient-specific feedback and healing responses. With these innovations, personalized medicine is set to transform chronic wound management into an evidence-driven, predictive, and patient-centered field that systematically reduces complications and accelerates the path to healing.

## Conclusion

The convergence of materials science, biomedical engineering, and cell biology has ushered in a new era for regenerative medicine, significantly advancing wound management and tissue regeneration. This review highlights how modern cell-engineered technologies are revolutionizing traditional approaches, offering more effective and personalized strategies for repairing damaged tissues. Over recent decades, regenerative medicine has made substantial strides in wound care through the integration of cell-engineered technologies, leveraging the versatile roles of cellular components like fibroblasts, keratinocytes, endothelial cells, and various stem cell populations. These cells are crucial in modulating inflammation, inducing angiogenesis, and restoring the extracellular matrix, all vital for effective healing. Therapeutic approaches using engineered cells, including MSCs, adipose-derived stem cells, bone marrow-derived progenitors, fibroblasts, and keratinocytes, have shown great promise in enhancing tissue repair, epithelization, and collagen deposition. Furthermore, advanced techniques incorporating biomaterials such as hydrogels, biodegradable scaffolds, 3D bioprinting materials, and diverse nanomaterials are redefining wound management by providing structural reinforcement, bioactive cues, spatial control, and sustained release of therapeutic biomolecules. Despite these promising advancements, challenges like immune response, cell survival post-transplantation, clinical outcome variability, and stringent regulatory oversight must be addressed. The future of wound care lies in personalized strategies, smart biomaterials, genetically modified cells, and the integration of cell engineering with AI, bioinformatics, and wearable devices. Establishing standardized manufacturing guidelines, long-term biosafety data, and cost-effectiv,e scalable models, alongside strong collaborations among stakeholders, is crucial to accelerate clinical translation. Cell-engineered technologies hold immense promise to become the cornerstone of advanced wound management, significantly improving patient recovery and quality of life.

## Data Availability

No datasets were generated or analyzed during the current study.
